# Additive manufacturing titanium components with isotropic or graded properties by hybrid electron beam melting/hot isostatic pressing powder processing

**DOI:** 10.1038/s41598-019-40722-3

**Published:** 2019-03-11

**Authors:** E. Hernández-Nava, P. Mahoney, C. J. Smith, J. Donoghue, I. Todd, S. Tammas-Williams

**Affiliations:** 1 Department of Materials Science and Engineering, University of Sheffield, Sheffield, S1 3JD UK; 2 School of Materials, University of Manchester, Manchester, M13 9PL UK; 3 Department of Maritime and Mechanical Engineering, Liverpool John Moores University, Liverpool, L3 3AF UK

## Abstract

A methodology has been demonstrated to consolidate Ti-6Al-4V powder without taking it to the liquid state by novel combination of the electron beam melting additive manufacture and hot isostatic pressing processes. This results in improved static mechanical properties (both strength and yield) in comparison to standard EBM processed material. In addition, the ability to generate microstructurally graded components has been demonstrated by generating a component with a significant change in both microstructure and mechanical properties. This is revealed by the use of electron backscattered diffraction and micro hardness testing to produce maps showing a clear distinction between materials consolidated in different ways. The variation in microstructure and mechanical properties is attributed to the different thermal history experienced by the material at different locations. In particular, it is found that the rapid cooling experienced during EBM leads to a typical fine α lath structure, whereas a more equiaxed α grains generated by diffusion is found in HIP consolidated powder.

## Introduction

Additive manufacturing (AM) has had a significant impact on metallurgical research in both academia and industry in recent years^1–3^. A wide range of AM techniques have become available and are united by the basic principles of depositing material layer by layer to produce components^1^. The advantages of AM are well documented in the literature^1,2^, so an exhaustive list is not provided here. For producing new parts with high spatial resolution, powder bed fusion (PBF) is commonly applied. PBF-AM involves layers of powder spread by a rake or wiper, which are then melted with a laser or electron beam into the geometry defined by a sliced CAD model.

Unfortunately, AM and PBF are not without drawbacks, not least their relatively slow deposition speeds and higher costs per unit manufactured compared to established manufacturing routes. Another issue is the surface finish of components, which is generally too poor for them to be used without some kind of finishing operation to reduce the surface roughness^4^. In addition, many alloys, when processed by standard AM melt strategies, have a microstructure dominated by columnar grains that grow through multiple layers^5,6^. Unsurprisingly, this can lead to anisotropic mechanical static and dynamic properties^7^. The final key drawback for PBF-AM is the potential for porosity formation. Porosity can be generated during PBF from a range of sources depending on the line energy of the heat source, ranging from simple lack of fusion between particles, to keyhole pores^8^. For example, keyhole porosity resulting from the vaporisation of low melting point elements in prealloyed powder^9^, has been reported to decrease the mechanical properties of AM components^10^. In addition, gas trapped within the powder feedstock can be retained within the consolidated material^11^. All pores act as stress concentrators, and the fatigue performance of material can be strongly influenced by their location and size^12^. Even more detrimental features such as cracks can result if the parameters are not optimised for the material being processed^13^. The high local cooling rates experienced during AM mean that most AM processes tend to produce parts with large residual stresses that must be heat treated prior to being put into service^7^.

One particular PBF technology, electron beam melting (EBM), minimises residual stresses by conducting the entire manufacturing cycle at an elevated temperature. Arcam AB (Gothenburg, Sweden) are currently the only suppliers of EBM equipment, and use proprietary software and a thermal model to maintain an elevated powder bed temperature throughout the build. Prior to melting each layer of powder, the electron beam is defocused and swept across the bed at a high speed. This both preheats and partially sinters the powder^14^. The sintering is necessary to avoid the build-up of negative charge where the electron beam interacts with the powder. By varying the level of preheat the bed temperature is controlled by the software. For Ti-6Al-4V the intention is to maintain the bed at 650 °C, which is high enough to reduce the residual stresses in built parts to near zero^15^. This removes the need for a stress relief heat treatment following manufacture, typically required for selective laser melted (SLM) parts.

In an attempt to avoid pores impacting the mechanical property of AM components, parts are often subjected to a hot isostatic pressing (HIPing) cycle prior to being put into service. The application of high temperatures and pressures causes pores to collapse and the newly contacting surfaces to bond by diffusion^16^. However, a concurrent coarsening of the microstructure leads to a reduction in yield strength of the material^17^.

To overcome some of the disadvantages of PBF-AM listed above, a hybrid approach to manufacturing has been proposed and demonstrated for SLM^18–20^. In particular, using SLM to generate a hollow preform before filling with powder and HIPing to consolidate the particles. Reducing the melt volume required leads to a commensurate decrease in build time for the AM process, enhancing its commercial viability. In addition, the columnar microstructure associated with melting and solidification can be avoided. Thus, two issues with AM have been at least partially addressed, the directional microstructure, and the high cost of manufacture.

While these benefits alone make the proposed method worthwhile of further exploration, other advantages may result in even greater possible positive outcomes. Particularly, the ability of the technique to generate material with site-specific properties and microstructures. By changing the properties with position, more efficient engineering structures could be generated than is currently possible^21^. For example, EBM has been used to generate lattice structures, where via spatial alteration of the lattice size and morphology, materials with better fatigue strength and energy absorption than standard uniform lattice structures, were achieved^22^.

In this paper, we report on how this hybrid approach to manufacturing can be applied to EBM AM, and report the microstructure and mechanical properties of Ti-6Al-4V produced by this approach. These are contrasted with Ti-6Al-4V manufactured by standard EBM techniques. Furthermore, we have demonstrated how it is possible to generate a microstructurally and mechanical property graded component by combining HIP and EBM.

## Experimental Methods

All samples were manufactured using an Arcam A2, with some subjected to further HIP processing. The pre-alloyed Ti-6Al-4V powder used for this study had been recycled a number of times, but the chemical composition remained within the allowable limits. The virgin powder was stated by the manufacturer to contain in weight percent: 6.45 Al; 3.94 V, etc., with powder batch number P1250. Over time it is likely that the oxygen level in the powder will increase. Chemical analysis of feedstock powder just prior to manufacture of the samples in the study showed oxygen content of 0.144 weight percent in accordance with ASTM F2924. Standard Arcam parameters recommended for Ti-6Al-4V and a layer thickness of 50 µm were used to manufacture all samples. In brief, the Arcam EBM process is conducted in four stages. Prior to material deposition, the system was taken to a high vacuum before backfilling with helium to a pressure of 2 × 10^−3^ mbar. As per standard Arcam operating procedures, a stainless steel baseplate was preheated to 730 °C by rapidly scanning it with the electron beam at a high power and low focus. The temperature was measured using a thermocouple below and in contact with the 10 mm thick baseplate. Throughout the build the Arcam software will attempt to keep the energy input at the correct level to ensure that the build temperature remains at around 650 °C. The baseplate was incremented downwards (*z*) in steps of 50 µm and powder spread across it with a rake, which traverses in the horizontal (x) direction. Again, the beam was applied with a high speed, low focus and high power, this time to preheat and sinter the powder to increase its electrical and thermal conductivity^14^. The sintered powder could then be safely melted by increasing the beam focus to produce small, rapidly moving melt pools, which first outlined the desired 2D geometry (termed contouring) before infilling the central areas (hatching). Standard Arcam recommended settings were used to manufacture all samples, and more detailed descriptions of the EBM process are available elsewhere^11,23^.

**Figure 1:**
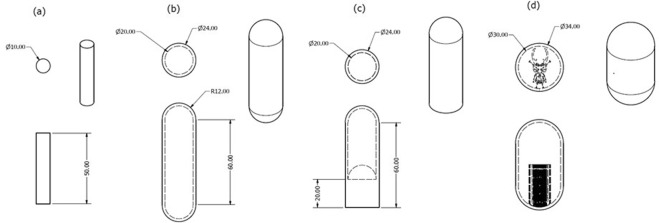
Geometries of samples manufactured using the EBM process: (**a**) solid samples melted with standard Arcam settings. Eight were manufactured in the x-direction and eight manufactured in the y-direction, four of each direction were HIPed prior to analysis. (**b**) Capsules where only the 2 mm outer skin was melted; four were manufactured in the x-direction and four in in the y-direction. (**c**) Sample containing both melted and unmelted powder, again four of each direction were manufactured. Finally, a single sample was manufactured with its long axis parallel to the build direction where some of the internal area was melted in the shape of a beetle.

Samples were manufactured in order to examine both the properties of the material produced by the standard EBM process, both as built and after HIP, and the EBM-HIP hybrid proposed here. Sixteen solid cylindrical specimens (geometry defined in Fig. 1a) were built to provide a baseline of standard EBM properties. Eight samples were orientated with their length aligned with the rake transverse (*x*) direction and eight with their length in the build (*z*) direction. The cylindrical samples in the horizontal direction were raised above the baseplate and used standard supports generated in Magics 20 (Materialise), whereas the vertical samples were built directly onto the baseplate. Four samples of each orientation were left in the as-deposited condition and four of each orientation were HIPed.

To examine properties of HIP consolidated powder, cylindrical capsules were built where only an outer 2 mm thick ‘skin’ was melted (Fig. 1b). The encapsulated powder remained in the as-sintered condition typical of the Arcam process. Again, samples were manufactured orientated in the build (z) and rake (x) direction. All samples were built offset from the baseplate on supports automatically generated in Magics 20. A second set of capsules was manufactured with half of the internal area melted by the electron beam (Fig. 1c).

In order to test the ability of the technique to manufacture complex graded geometries and how distinct the boundaries between the two manufacturing techniques were, a stylised 2D image of a beetle was extruded upwards to generate a CAD model. This was then enclosed in a 2 mm skin (Fig. 1d), and orientated in the EBM build such that stag beetle image was normal to the build direction. During EBM processing, the beetle shape was melted while the surrounding powder remained un-melted.

HIPing was conducted by Bodycote H.I.P. Ltd (Chesterfield, United Kingdom) using the standard parameters developed to heal porosity in cast Ti-6Al-4V parts. This consisted of heating at 8.8 K/minute up to a 2-hour hold at 1193 K (920 °C) and 100 MPa, followed by cooling to room temperature at 7.8–8.5 K/minute.

To avoid repeated description of the various processing conditions experienced by different Ti-6Al-4V samples, three designations will be used hereafter. Ti-6Al-4V produced by standard Arcam EBM melt strategies is denoted EBM. Ti-6Al-4V that is first melted during the EBM build but then subjected to the HIP cycle is labelled EBM-H. Finally, the encapsulated sintered powder consolidated during the HIPing is labelled EBS-H. When the interface between EBM-H and EBS-H (Fig. 1c) was tensile tested these samples are labelled EBM/S-H.

One of each of the geometries/orientations/conditions shown in Fig. 1a–c was retained for density determination and hardness testing, whereas the other three samples were used to examine the tensile properties. Special Testing Ltd (Sheffield, United Kingdom) machined and tested the material in accordance with ASTM A370 2017 using a 4 mm gauge diameter. Care was taken during tensile specimen machining of EBS-H samples to ensure that the outer 2 mm skin was removed to test only the consolidated powder within. Fractography of the broken tensile specimens was conducted with scanning electron microscopy (SEM) imaging using a FEI-Inspect F microscope with 15 kV of accelerating voltage, a spot size of 3 µm and a working distance of 10 mm.

For microstructural analysis and density determination planes normal to the longest length of each as built sample were prepared using standard metallurgical techniques to produce a mirror finish, in line with other work in the literature^5^. High resolution imaging of the grain morphology and EBSD analysis of the orientations within the beetle capsule (Fig. 1d) was performed with a Zeiss Sigma SEM, whereas a lower resolution, but large area mapping was carried out with a TESCAN MIRA3. Imaging was carried out in backscattered electron mode to determine any microstructural variation between consolidation methods, and coarse scale EBSD was used to gather information about the crystallographic orientations and their distribution across the build. Two EBSD maps were gathered, using an Oxford Instruments NordlysNano EBSD detector paired with AZtec acquisition software. A larger map, with a 5 µm step size, was used to image an area of 25.3 mm by 9.3 mm, almost the entire cross section. A smaller, higher resolution map, an area of 5.5 mm by 4.2 mm with a 2.5 μm step size was used to calculate pole figures for the two different phases. EBSD analysis was carried out using HKL Channel 5, and the high temperature prior β microstructure calculated from the room temperature α orientation measurements using software developed at the University of Sheffield by Davis and Wynne that utilises the Burger’s orientation relationship^24^.

For density determination, planes normal to the testing direction (Fig. 1a–c) were examined as polished, i.e. unetched. Optical micrographs were collected using an Olympus microscope equipped with Clemex software, which allows the stitching of multiple images to provide large, high-resolution images of each direction and condition. 36 images were taken of the central region of each sample at 200x magnification, resulting in a total imaged area of 10.9 mm^2^. The relative density of the specimens were quantified by employing the Otsu method^25^ to segment the data into ‘solid’ and ‘void’ in MATLAB. Higher resolution, qualitative evaluation of individual pores was conducted with an InspectF SEM equipment previously described.

The hardness of samples was investigated using a Struers durascan 70 automated indenter. The polished planes used for density determination for each condition (i.e. EBM, EBM-H and EBS-H samples) were indented with 1 kg in 5 × 5 arrays with 0.5 mm spacing. In addition, the hardness of the beetle capsule was investigated using two 0.5 mm spaced 51 × 24 HV2.5 indent arrays (diagonally offset and overlaid) in order to identify the any functional changes regarding the materials mechanical performance. Test conditions were chosen to provide a resolution high enough to pick up local changes in hardness over a testable number of indents.

## Results

**Figure 2:**
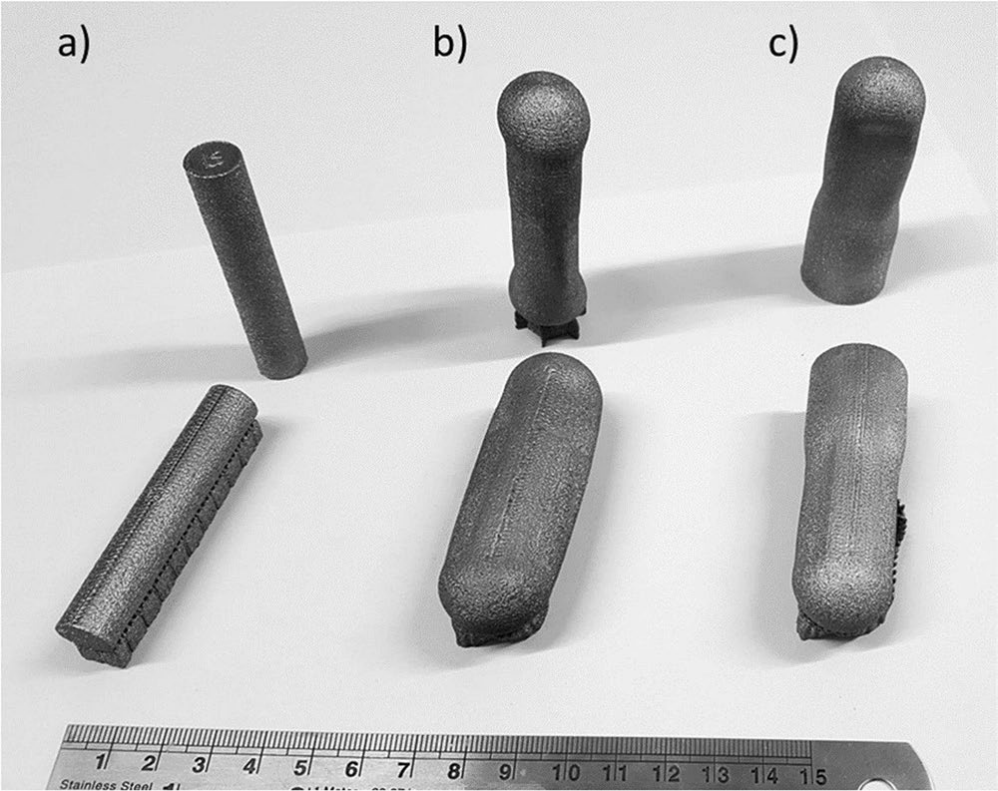
Photograph of samples following HIPing, with alphabetical designations corresponding respectively to (**a**) EBM-H, (**b**) EBS-H, and (**c**) EBS/EBM-H.

Following HIPing, visual examination of the samples with encapsulated sintered powder revealed that there had been a significant decrease in volume, whereas no change was apparent in the EBM consolidated samples that were HIPed following manufacture. In addition, the solid skin had buckled in some locations as exemplified in Fig. 2b,c.

### Microstructural observations in graded material

The “beetle” sample was used to examine both EBM-H and EBS-H material, and the structure of the graded materials. The microstructure of standard EBM TI-6Al-4V was not examined in detail due to the number of already existing studies (e.g. refs.^5,6,17^).

#### Grain morphology

**Figure 3:**
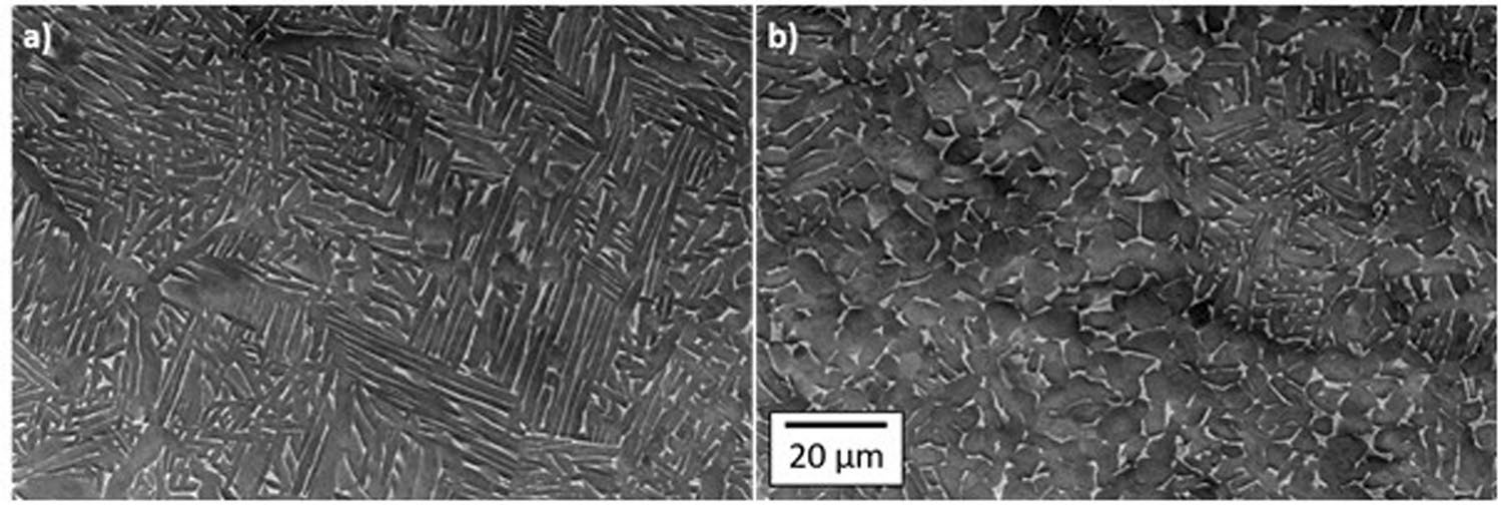
Example SEM micrographs taken from regions within the beetle sample (**a**) EBM-H, and (**b**) EBS-H. Here the build direction is normal to the plane of the page. Both images to same scale.

Backscatter SEM images of the beetle section revealed differences in microstructure (Fig. 3), between regions. There was no visible void of boundary between the two regions implying good bonding between the two. The EBM-H material consisted of α laths transformed from prior β grains with both Widmanstätten and colony morphologies present, and fine residual β remaining between laths. In contrast, the EBS-H regions were primarily composed of α with a morphology much closer to equiaxed and coarser β primarily at α grain boundary triple points.

**Figure 4:**
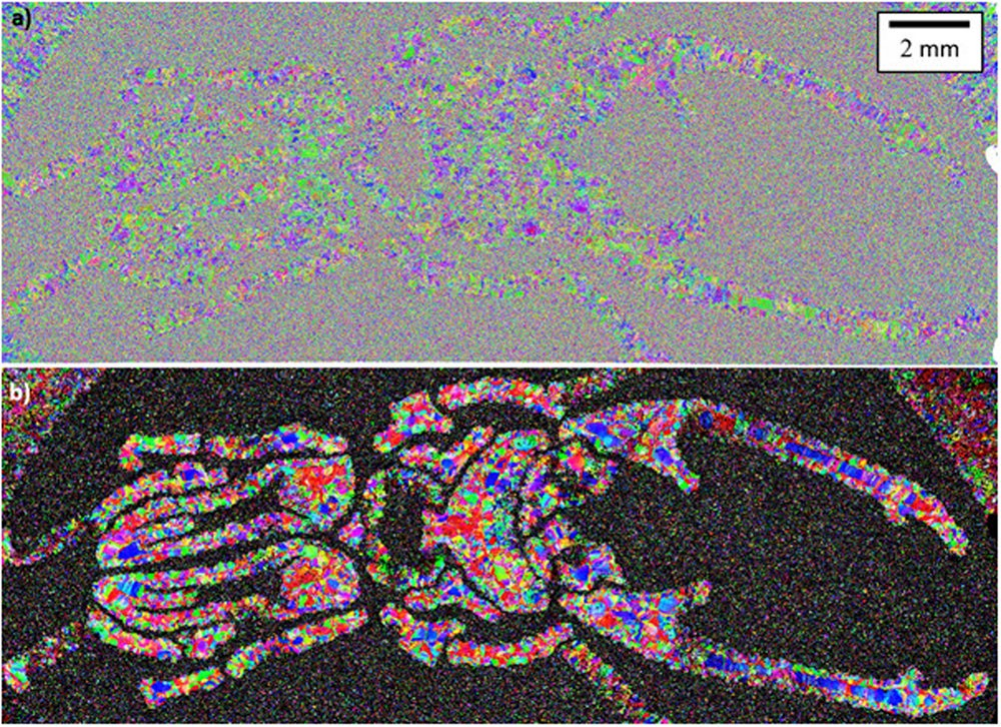
Grain orientation data regarding the beetle capsule. (**a**) Room temperature α phase grain orientation information collected by EBSD from the beetle sample; (**b**) Initially solidified β microstructure reconstructed from, and at the same scale as, data in (**a**). Here, the build direction (z) is normal to the plane of the page.

An EBSD map of the entire beetle is shown in Fig. 4a, providing information about the orientations of grains. Visual inspection of the data is sufficient to identify the beetle shape, albeit slightly distorted. In addition, the melted skin of the capsule is also visible in the upper corners of the map. The number density of grain boundaries is higher in the EBS-H region in comparison to the EBM-H. Indeed, it appears that the EBM-H areas have colonies of multiple similar orientated laths, which appear as a single grain due to the resolution of the EBSD not being able to detect the thin β between the laths.

The boundary between the EBM-H and EBS-H regions becomes much more visually apparent when software is used to calculate the high temperature β grain structure from which the α phase transformed (Fig. 4b). This is primarily due to the extent with which the reconstruction software was able to calculate the β phase grain orientations. In the EBM-H regions, large β grains can be observed. In contrast, in much of the EBS-H region the software was unable to determine the β phase orientations resulting in larger ratio of unidentified (black) points. The β grains that the software was able to identify within the EBS-H region have a much smaller average size than those in the EBM-H region. However, the number of non-indexed points, and there being too few points in each grain, means that reliable grain size statistics could not be produced.

#### Texture

**Figure 5:**
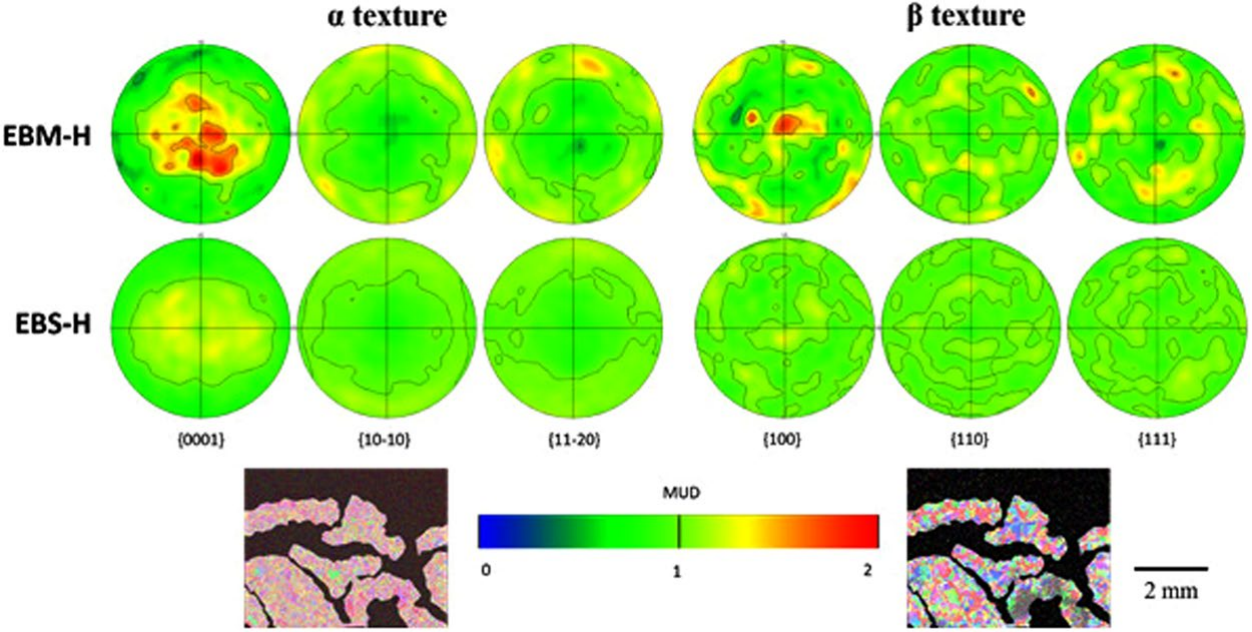
Pole figures obtained from EBSD maps of α phase (left) and reconstructed β (right). The EBSD maps used to generate the pole figures are shown in the insect images (both to same scale) with material classified as EBM-H highlighted.

It is immediately apparent from inspection of the pole figures (Fig. 5) generated from the α phase EBSD maps (inset) that the crystallographic texture of the two regions also showed some considerable differences. The EBM-H regions melted by the electron beam had a maximum-recorded multiple of uniform density (MUD) of 2.1, whereas the EBS-H regions showed a maximum of 1.3. It is clear that the EBS-H region was less textured than the EBM-H region, however, there was still some observable departure from a completely random structure.

A similar trend was observed when the initial solidification β grain structure was reconstructed, and used to plot pole figures (Fig. 5). In particular, a MUD of 1.8 was recorded in the EBM-H region in comparison to 1.3 in the EBS-H region.

### Density measurements and defect morphology

All of the samples were found to contain porosity. However, there were differences in the pore volume fractions measured, with the highest in the standard EBM material (0.069%), followed by the EBS-H (0.020%), and the lowest in EBM-H (0.005%).

**Figure 6:**
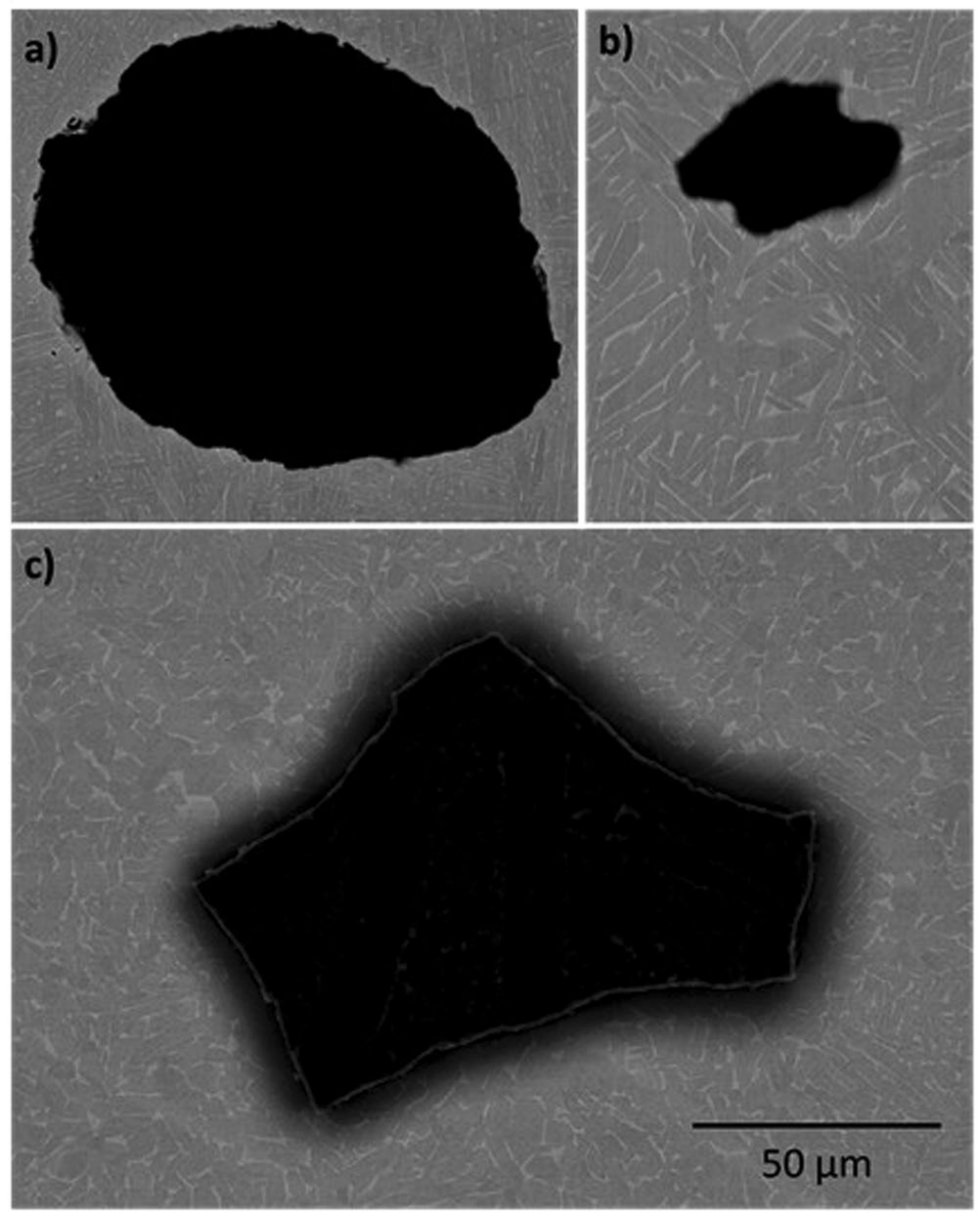
SEM images of examples of larger porosity found in (**a**) EBM, (**b**) EBM-H, and (**c**) EBS-H. All images to same scale.

One of the largest pores found in each condition is shown in Fig. 6. EBM material contained mainly round pores, with fewer more irregular voids also observed. The pores observed in the EBM-H material were smaller and less regular. It is notable that of the planes observed, the largest pore was found in EBS-H. This pore (Fig. 6c) was also irregular in morphology, although many of the other pores in this material were close to circular.

### Mechanical properties

#### Hardness

**Figure 7:**
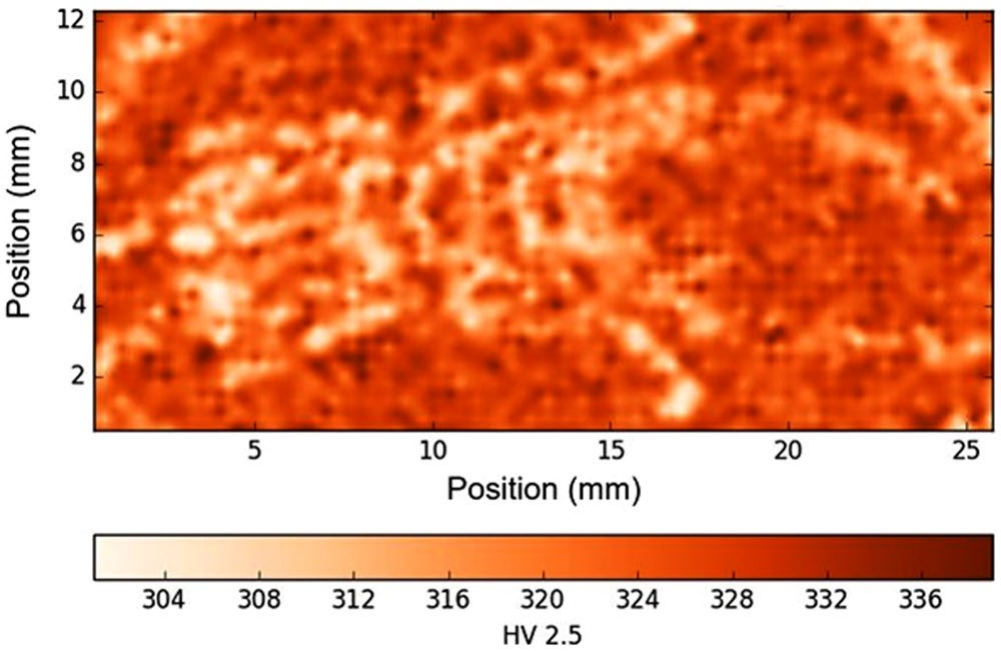
Macro hardness map of beetle sample. Here, the build direction (z) is normal to the plane of the page.

The hardness map presented in Fig. 7 corresponds to material previously shown in the EBSD map in Fig. 4. It clearly demonstrates the variation in measured hardness between the EBM-H and EBS-H consolidated regions in polishing plane of the beetle sample. Similar to the EBSD map, the beetle shape melted during EBM is clearly visible, although distorted in the same way as the EBSD data. The softer melted skin of the capsule is also visible in the upper corners of the map. Areas melted during EBM processing tended to be softer than those densified during HIPing, with approximately 10% difference in recorded hardness.

**Figure 8:**
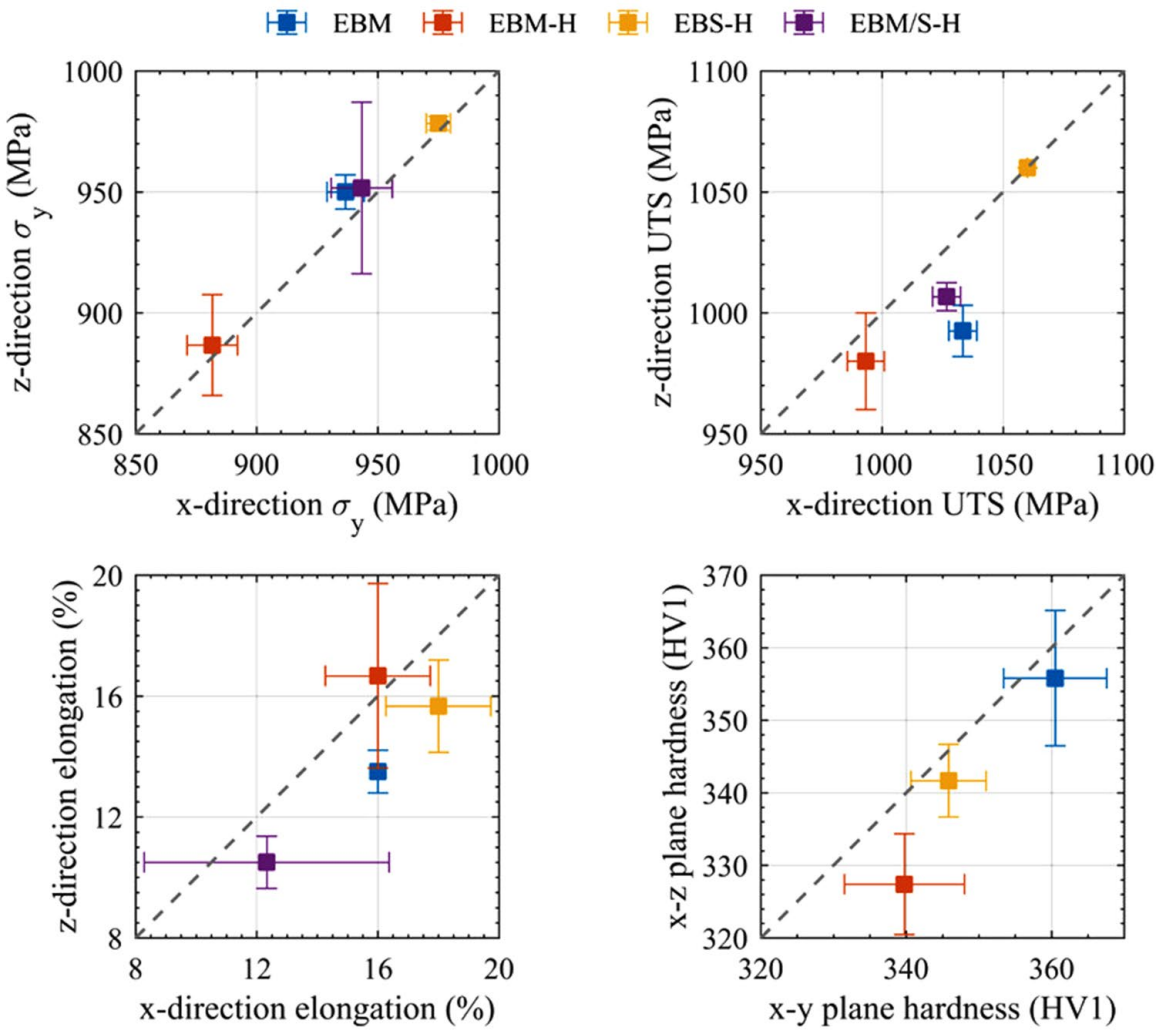
Comparison of data collected in this study showing mechanical property anisotropy. The mean value recorded in the x-direction is plotted against the mean value in the z-direction. The error bars indicate the standard deviation of 3 tensile tests in each condition and direction, for σ_y_, UTS and elongation, and 25 hardness tests. The dotted line indicates equivalence between testing directions, i.e. perfectly isotropic behaviour.

The average and standard deviation recorded after 25 hardness tests in the polished planes of the tensile blanks used for density determination is shown in Fig. 8. All samples were found to be slightly harder in the *x-y* plane than the *x-z* plane, with EBM showing the highest hardness, and EBM-H the lowest.

#### Static tensile testing

**Figure 9:**
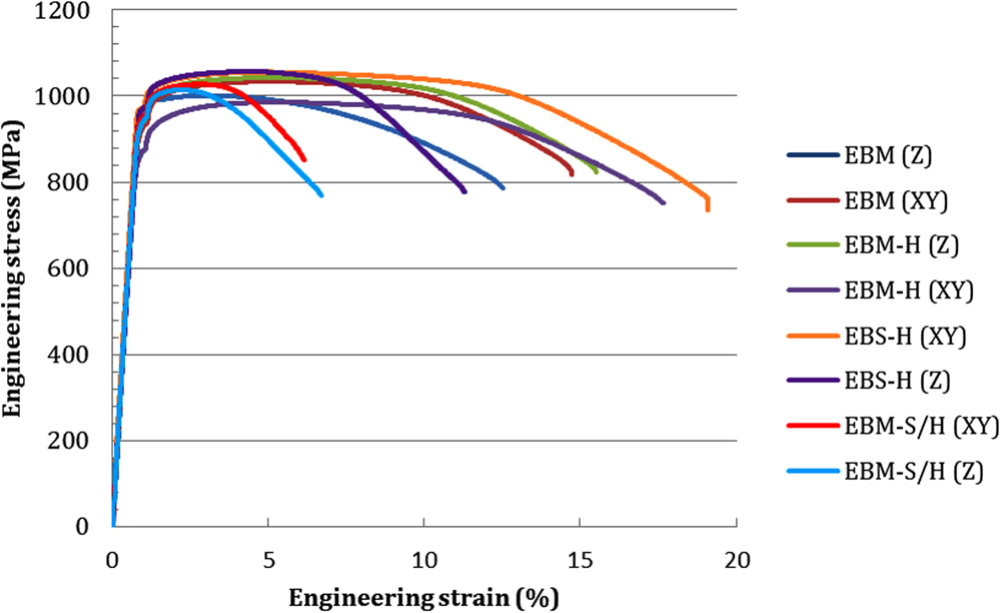
Example stress-stain plots for all samples tested, colour indicates material condition and orientation.

In order to enable numerous results of different material conditions and testing directions to be quickly compared, the yield stress (*σ*_*y*_), ultimate tensile stress (UTS) and elongation to failure have been plotted in Fig. 8, while example stress strain plots for each condition are shown in Fig. 9. EBS-H material was found to have a higher *σ*_*y*_ and UTS than standard EBM, and EBM-H material lowest. When the interface between EBM-H and EBS-H (EBM/S-H) was tested, the results were found to be reduced below the EBS-H material, but similar to standard EBM. The scatter was also lower in the EBS-H in comparison to all other conditions, for both sigma-y and UTS. In fact, when the data was rounded in accordance with ASTM E29, all the tested EBS-H samples (6 total, 3 in × and 3 in z) were reported to have a UTS of 1060 MPa. The EBS-H is both highly consistent and isotropic. In contrast, EBM and EBM/S-H were found to have higher UTS in the x-direction than the z. The elongation to failures recorded displayed more scatter, but in general, the EBM-H and EBS-H showed the highest values, followed by the standard EBM and then the EBM/S-H.

**Figure 10:**
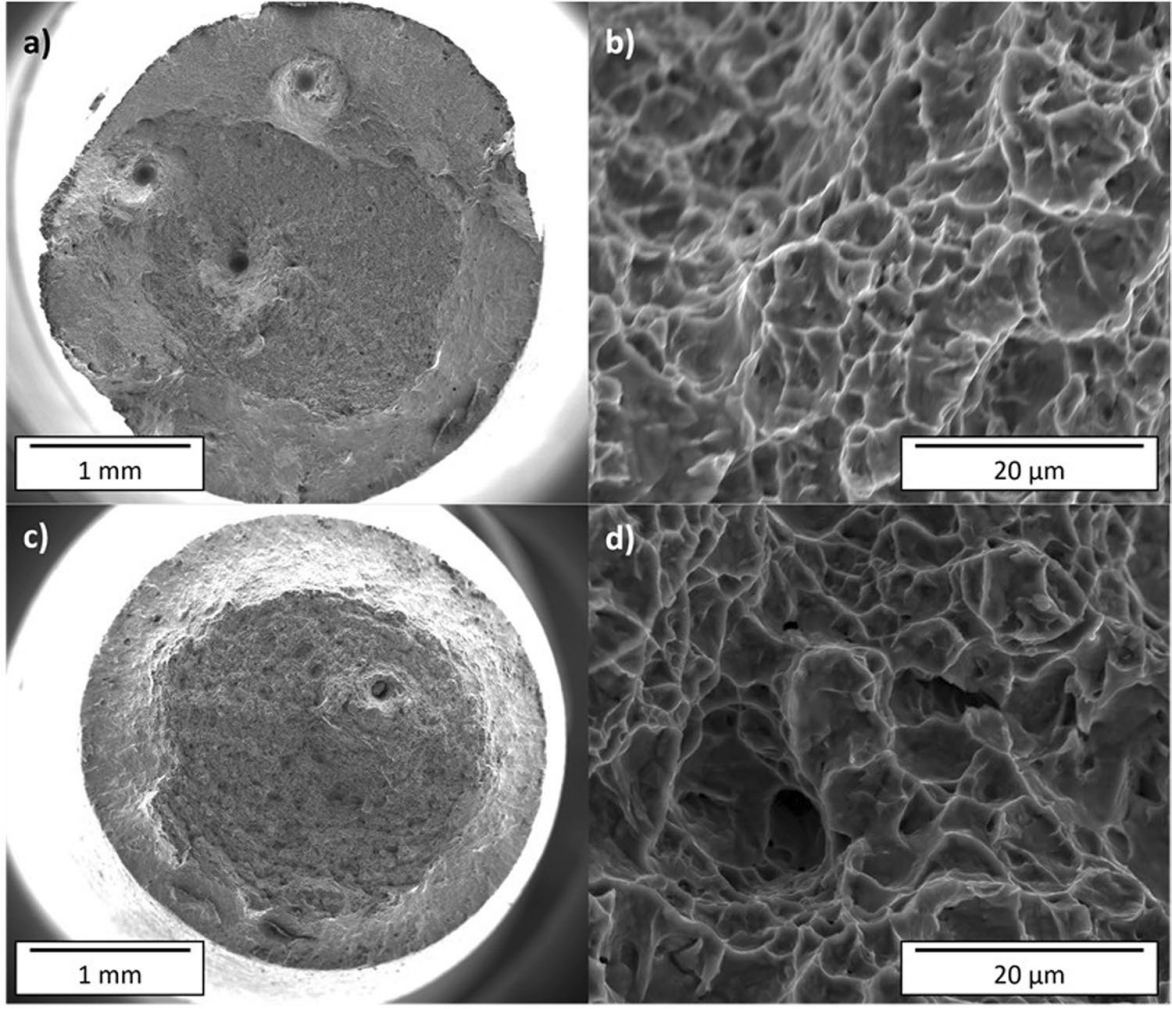
Fracture surfaces imaged by SEM of samples tested in the z-direction. Macrograph (**a**) and high resolution (**b**) images of EBM sample, and macrograph (**c**) and high-resolution (**d**) image of EBS-H sample.

Examples of EBM and EBS-H fracture surfaces are shown in Fig. 10. Defects are observable on all fracture surfaces, although a greater number are visible in the EBM material. Higher resolution images of the fracture surfaces revealed similar morphologies for all conditions and testing directions.

## Discussion

It has been demonstrated that it is possible to produce high density Ti-6Al-4V parts with graded microstructure and properties by combining the EBM and HIP process. It is clear is that the 2 mm outer skin of the capsules melted by the electron beam during the EBM process was impervious to the argon used during the HIPing process. The capsules thus acted in a manner analogous to a standard HIP can. That is, the capsules collapsed and provided the pressure on the powder to consolidate to a dense volume at the temperatures experience during HIPing. Furthermore, like a HIP can, the skin was removed by machining in order to reveal and assess the consolidated EBS-H material within.

### Densification of powder

From the results shown above it is apparent that the standard HIP cycle applied was sufficient to almost completely densify the sintered powder. Titanium powder sintered during the EBM process typically has a density of around 50–60%^14^. In contrast, examination of the EBS-H material revealed a density of >99.9%. During the HIP cycle, the powder particles will have been brought into contact and diffusion bonded, as per a standard powder HIP process. The thermophysical effects during HIPing have been well described by previous studies^26–28^.

It would be possible to manufacture whole components with this technique, or alternatively selectively melt regions to generate a graded microstructure (see below). Powder metallurgy HIP is well established method to create near net shape components with fine homogeneous microstructures^27^. Typically, this is achieved by welding HIP cans to create a preform skin, which is then filled with powder and HIPed. Finally, the skin is machined off to reveal the component beneath. By generating the skin using EBM, the labour costs will be significantly reduced. Furthermore, the build time of the EBM process will be reduced due to the lesser melt volume, reducing costs in comparison to standard EBM. Of course, unlike in standard EBM AM, it is essential that the part is HIPed for it to have any load bearing strength. However, given the strong dependence of the fatigue life on the presence of porosity/defects, any components that would experience cyclic load in service would likely be HIPed to improve their fatigue life. Thus, the HIP process may be adopted as standard procedure for AM components, and hence its use to consolidate the powder would not result in any additional cost.

Unfortunately, the increased complexity of part design may lead to higher pre-manufacture design costs. Standard AM parts can be directly produced from CAD models. Utilising the method proposed here requires significant CAD pre-processing, with both removal of the internal melt area and scaling up of the part geometry to ensure that the reduced volume after consolidating the powder by HIP meets the needs of the original component. However, tools developed for standard powder metallurgy HIP (for example ref.^29^) could likely be used to assist with part design.

EBM takes place in a vacuum chamber and is thus more naturally suited to this technique than some competing technologies, such as SLM, which take place under an inert argon atmosphere at approximately 1 atm. Using SLM to produce the capsule would lead to argon becoming trapped between powder granules within the impermeable skin. Given that argon is used to apply the pressure during HIPing it is clear that this internal argon must remain within skin during HIPing, but, as the volume deceased, higher pressure. Thus, there is the possibility that these small, high-pressure pockets of argon may expand in the future. In fact, this phenomenon has been exploited to generate metallic foams by heat treating HIP densified powder with argon deliberately left between the particles, which leads to large expansion of the argon bubbles by plastic deformation^30,31^. Both SLM and EBM could potentially be affected by the presence of argon bubbles trapped within the powder feedstock. After HIPing the argon within powder would remain and could potentially regrow if the HIPed part was heat treated at a high enough temperature. Indeed, such effects have been observed by other authors when heat treating standard powder HIP Ti-6Al-4V^32^. The effect of this on the mechanical property requires scrutiny.

While the EBM process does not happen in a high vacuum, but at controlled vacuum of 2 × 10^−3^ mbar, the relatively low helium pressure would lead to very limited gas entrapment in comparison to SLM. In contrast to argon filled SLM pores, these helium pores would contain a much lower number of moles of atoms, due to the significantly reduced pressure when they were first entrapped. As such, upon HIPing the volume of gas could become much smaller before reaching equilibrium with HIPing pressure. They would thus be unlikely to regrow to the same extent as the argon filled pores introduced at atmospheric pressure during SLM.

### Microstructure and mechanical properties

While EBM of Ti-6Al-4V is a relatively well-established AM technique, we have shown the static mechanical properties can be improved by harnessing the EBS-H methodology proposed here. The EBS-H Ti-6Al-4V was found to have higher yield, UTS and elongation than standard EBM Ti-6Al-4V, exemplifying the benefits of this modified manufacturing route. In addition, the reduced scatter in results may allow smaller safety factors to be used and give engineers the ability to maximise the efficiency of the material. While it appears that the mean values of EBS-H are closer to isotropic than standard EBM, the scatter in both datasets makes it hard to draw definitive conclusions. In particular, for all conditions the difference from isotropic behaviour is not statistically significant, as has been found by a number of other studies of EBM material^33^.

The grains within the EBS-H material appear to have a size greater than the width of the lathes in the EBM-H material (Fig. 3), which would suggest according to a simple application of the Hall-Petch relationship that the material would have a lower yield stress. However, as exemplified in the EBSD data, the colonies are significantly larger in the EBM-H than the grains in EBS-H. The colonies can allow slip through multiple laths and thus colony size can be correlated with mechanical performance^34^. Hence, the larger colonies lead to the lower yield stress recorded for the EBM and EBM-H compared to EBS-H.

The α grains in the EBS-H material appears more equiaxed than those in the EBM or EBM-H, which appear as fine and slightly coarser laths respectively^17^. This can be attributed to the differing routes by which the microstructure develops. The development of the typical EBM Ti-6Al-4V microstructure has been described by a number of authors^5,6,17^. In brief, the rapid cooling of initially solidified coarse columnar β grains through the β transus leads to a fine Widmanstätten microstructure. Subsequent HIPing of EBM leads to a coarsening of the microstructure due to growth at the high temperatures experienced during HIP^17^. In contrast, when titanium powder particles are compacted and densified during HIP the material remains below the β-transus and consolidation occurs by diffusion.

Contrary to the results presented here, previous studies of Ti-6Al-4V consolidated from powder HIP have observed a higher aspect ratio, lath morphology of the grains^28,35^. Subsequent heat treatments were found to an increase the measured fraction of equiaxed grains, proportional to treatment time and temperature^35^. The mostly equiaxed α grains observed in the HIP consolidated powder analysed in this paper (EBS-H) is thus more typical of heat-treated HIP consolidated powder. While there was no heat treatment following the HIP for the EBS-H material it is important to note that the powder was held at high temperature during the initial EBM sample manufacture for a significant period of time. In addition, this powder had been recycled a number of times so it is difficult to ascertain exactly how long the powder had been held at high temperature other than to say it is likely that much of it had experienced tens of hours above 700 C. At this high temperature α grains within the powder may have begun to spheridise during the EBM build, prior to the HIP. This spheroidisation may remain following EB sintering and HIP and could thus account for the larger fraction of equiaxed α that was observed in EBS-H than has been found by previous authors.

A note of caution must be made about the potential fatigue properties of the EBS-H Ti-6Al-4V, and the potentially detrimental pores that did remain in the EBS-H material. In particular, the large irregular pore shown in Fig. 6c, which has a morphology suggesting it originated at the interface between powder particles. Unfortunately, such large irregular defects would likely have a significant effect on the fatigue life due to the stress concentration they generate^12,36^. However, there is scope to modify the HIP cycle to try to avoid such defects appearing^28^. In addition, the sintering step during EBM could be modified to create a more dense sintered volume prior to HIPing which may aid attempt to reduce porosity in EBS-H material.

### Materials with site-specific properties

The EBM-HIP method demonstrated in this paper has the potential to produce either entire components of close to isotropic properties or a graded microstructure where only some of the component is melted. Graded material has the potential to allow significantly improved component design by tailoring the properties to the conditions experienced in different locations^11^.

The tensile testing conducted here suggests there is no reduction in yield/UTS associated with the interface between EBM/S-H in comparison to pure EBM-H. Thus, designers can generate CAD models employing materials with site-specific properties, where the local material characteristics are optimised for the local conditions, but with no reduction in strength associated with the overlap. However, the reduction in ductility may be of concern.

It is notable that should this technique be applied to other alloys, it may be possible to generate an even greater change in properties. The titanium alloy used in this study was chosen due to the availability of literature, feedstock materials, and process parameters for both AM and HIP cycles. However, the initially solidified β rapidly transforms to fine α laths as the material cools below the β transus. Thus, the potential influence of the columnar grains on mechanical properties is reduced. In contrast, in other engineering alloys the columnar grains could be retained, allowing the possibility of a greater duality in the microstructure and properties. For examples, columnar regions could be used to increase the resistance in creep in selected regions, while more equiaxed areas could provide a higher yield stress in others.

## Conclusions

A technique has been demonstrated to generate material with better static mechanical properties than standard EBM Ti-6Al-4V. This was achieved by using EBM AM to generate a hollow preform filled with sintered powder, before HIPing to full density. The improvement in properties was attributed to a more equiaxed microstructure, which has a smaller grain size than the effective grain (colony) size of EBM Ti-6Al-4V. Importantly, by selectively melting some of the internal section of the samples, a graded microstructure can be generated. A complex graded microstructure was manufactured and changes in grain morphology and orientation were found to closely align with the intended geometry. A reduced but not eliminated texture was observed in the HIP consolidated powder in comparison to melted powder, and mechanical tests revealed behaviour that was closer to isotropic. However, the limited data available requires further testing to collaborate our results.
